# Topical issue on Lattice Field Theory during the Covid-19 pandemic

**DOI:** 10.1140/epja/s10050-021-00614-5

**Published:** 2021-12-09

**Authors:** F. Knechtli, T. Luu, C. Urbach

**Affiliations:** 1 Department of Physics, Bergische Universität Wuppertal, Gaußstr. 20, 42119 Wuppertal, Germany; 2 Institut für Kernphysik 3, Institute for Advanced Simulation 4, Forschungszentrum Jülich, 54245 Jülich, Germany; 3 Helmholtz-Institut für Strahlen- und Kernphysik and Bethe Center for Theortical Physics, Universität Bonn, 53115 Bonn, Germany

Like many other scientific conferences scheduled during the year 2020, the *International Symposium on Lattice Field Theory 2020* (in short Lattice 2020)—originally planned to take place at the University of Bonn, Germany, coorganised with Forschungszentrum Jülich and the University of Wuppertal—was cancelled due to the Covid-19 pandemic. Under normal circumstances this annual conference allows researchers in the field to meet in person, exchange ideas, and discuss their most recent research topics. It also provides an opportunity for giving presentations and the corresponding proceeding contributions serve an important service to the community by providing concise, summarised reports that help quantify the progress of the field over one year. Equally important, the review presentations at these lattice conferences offers an important opportunity for young academics in the field to introduce themselves to the community and to advance their careers. The Covid-19 pandemic through this all out the window.

After a seed workshop at CERN in 1982, the series of lattice conferences started in 1984 as a three day meeting at Argonne and saw even two meetings in 1985 in Tallahassee and Wuppertal. Since then the conference has occurred annually and uninterrupted until the year 2020, with its location alternating between the Americas, Europe, and Asia. During this time the relatively small community has grown substantially, with now over 500 participants when the conference takes place in Europe and a little less in the Americas or Asia. Likewise, the spectrum of topics has widened. And while the focus is still mainly on lattice QCD and connected areas, the ubiquitous nature of lattice Monte Carlo means that there is always room for new developments, whether they be inspired by other fields of physics, or conversely, they are applied to other fields of physics.

This issue presents a set of review articles that aim to compensate for the aforementioned missed opportunities. It hopes to provide—at least to a small extent—the service usually provided by the review plenary presentations to the community. Since the international advisory committee and the local organising committee had already finalised the list of speakers by the time the conference had to be cancelled, these yet-to-be invited speakers were given the opportunity to prepare a review article.

The eight review articles that were received for this issue cover a wide range of topics relevant to the field and beyond. We hope that this set of reviews will be useful for the community and that they provide some sense of continuity to this important conference. We especially thank all authors and reviewers for their contributions, particularly during these difficult times.

Francesco Knechtli, Thomas Luu, and Carsten Urbach

Guest Editors

## Table of contents

1. *The anomalous magnetic moment of the muon: status of lattice QCD calculations* (A. Gérardin) [1].
2. *Overview of the QCD phase diagram – Recent progress from the lattice* (J. N. Günther) [2].
3. *The x-dependence of hadronic parton distributions: A review on the progress of lattice QCD* (M. Constantinou) [3].
4. *Quark flavour physics and lattice QCD* (M. Wingate) [4].
5. *Excited states in nucleon structure calculations* (K. Ottnad) [5].
6. *Flavor-diagonal CP violation: the electric dipole moment* (A. Shindler) [6].
7. *Past, present, and future of precision determinations of the QCD coupling from lattice QCD* (M. Dalla Brida) [7].
8. *The large* $N_c$ *limit of QCD on the lattice* (P. Hernández, F. Romero-López) [8].
