# Erratum to: Topical issue on Lattice Field Theory during the Covid-19 pandemic

**DOI:** 10.1140/epja/s10050-022-00691-0

**Published:** 2022-03-17

**Authors:** F. Knechtli, T. Luu, C. Urbach

**Affiliations:** 1 Department of Physics, Bergische Universität Wuppertal, Gaußstr. 20, 42119 Wuppertal, Germany; 2 Institut für Kernphysik 3, Institute for Advanced Simulation 4, Forschungszentrum Jülich, 54245 Jülich, Germany; 3 Helmholtz-Institut für Strahlen- und Kernphysik and Bethe Center for Theortical Physics, Universität Bonn, 53115 Bonn, Germany

## Erratum to: Eur. Phys. J. A (2021) 57:326 10.1140/epja/s10050-021-00614-5

In the original editorial paper, there was a typo in the title in the table of contents for refs. [1] and [7]. The corrected table of contents can be found below.

## Table of contents

1. *The anomalous magnetic moment of the muon: status of lattice QCD calculations* (A. Gérardin) [1].
2. *Overview of the QCD phase diagram – Recent progress from the lattice* (J. N. Günther) [2].
3. *The x-dependence of hadronic parton distributions: A review on the progress of lattice QCD* (M. Constantinou) [3].
4. *Quark flavour physics and lattice QCD* (M. Wingate) [4].
5. *Excited states in nucleon structure calculations* (K. Ottnad) [5].
6. *Flavor-diagonal CP violation: the electric dipole moment* (A. Shindler) [6].
7. *Past, present, and future of precision determinations of the QCD coupling from lattice QCD* (M. Dalla Brida) [7].
8. *The large* $N_c$ *limit of QCD on the lattice* (P. Hernández, F. Romero-López) [8].
